# Sequence-Based Prediction of Metamorphic Behavior in Proteins

**DOI:** 10.1016/j.bpj.2020.07.034

**Published:** 2020-08-14

**Authors:** Nanhao Chen, Madhurima Das, Andy LiWang, Lee-Ping Wang

**Affiliations:** 1 Department of Chemistry, University of California, Davis, California; 2 School of Natural Sciences, University of California, Merced, California; 3 Chemistry & Chemical Biology, University of California, Merced, California; 4 Center for Cellular and Biomolecular Machines, University of California, Merced, California; 5 Center for Circadian Biology, University of California, San Diego, La Jolla, California; 6 Quantitative & Systems Biology, University of California, Merced, California; 7 Health Sciences Research Institute, University of California, Merced, California

## Abstract

An increasing number of proteins have been demonstrated in recent years to adopt multiple three-dimensional folds with different functions. These metamorphic proteins are characterized by having two or more folds with significant differences in their secondary structure, in which each fold is stabilized by a distinct local environment. So far, ∼90 metamorphic proteins have been identified in the Protein Databank, but we and others hypothesize that a far greater number of metamorphic proteins remain undiscovered. In this work, we introduce a computational model to predict metamorphic behavior in proteins using only knowledge of the sequence. In this model, secondary structure prediction programs are used to calculate diversity indices, which are measures of uncertainty in predicted secondary structure at each position in the sequence; these are then used to assign protein sequences as likely to be metamorphic versus monomorphic (i.e., having just one fold). We constructed a reference data set to train our classification method, which includes a novel compilation of 136 likely monomorphic proteins and a set of 201 metamorphic protein structures taken from the literature. Our model is able to classify proteins as metamorphic versus monomorphic with a Matthews correlation coefficient of ∼0.36 and true positive/true negative rates of ∼65%/80%, suggesting that it is possible to predict metamorphic behavior in proteins using only sequence information.

## Significance

This article introduces the diversity index as a descriptor to distinguish metamorphic proteins, which possess multiple stable folds, from monomorphic proteins that possess only onefold. The diversity index is designed to measure uncertainty in computationally predicted secondary structure, which we hypothesize is elevated for metamorphic proteins. We tested our hypothesis by training a binary classifier using the diversity index and an annotated data set of metamorphic and monomorphic proteins and found an optimal Matthews correlation coefficient of 0.36, supporting the hypothesis and demonstrating for the first time, to our knowledge, that it is possible to predict metamorphic behavior in proteins using only sequence information. The sequence-based classifier has broader applicability compared to methods that rely on making comparison to experimentally measured structures.

## Introduction

**Figure 1:**
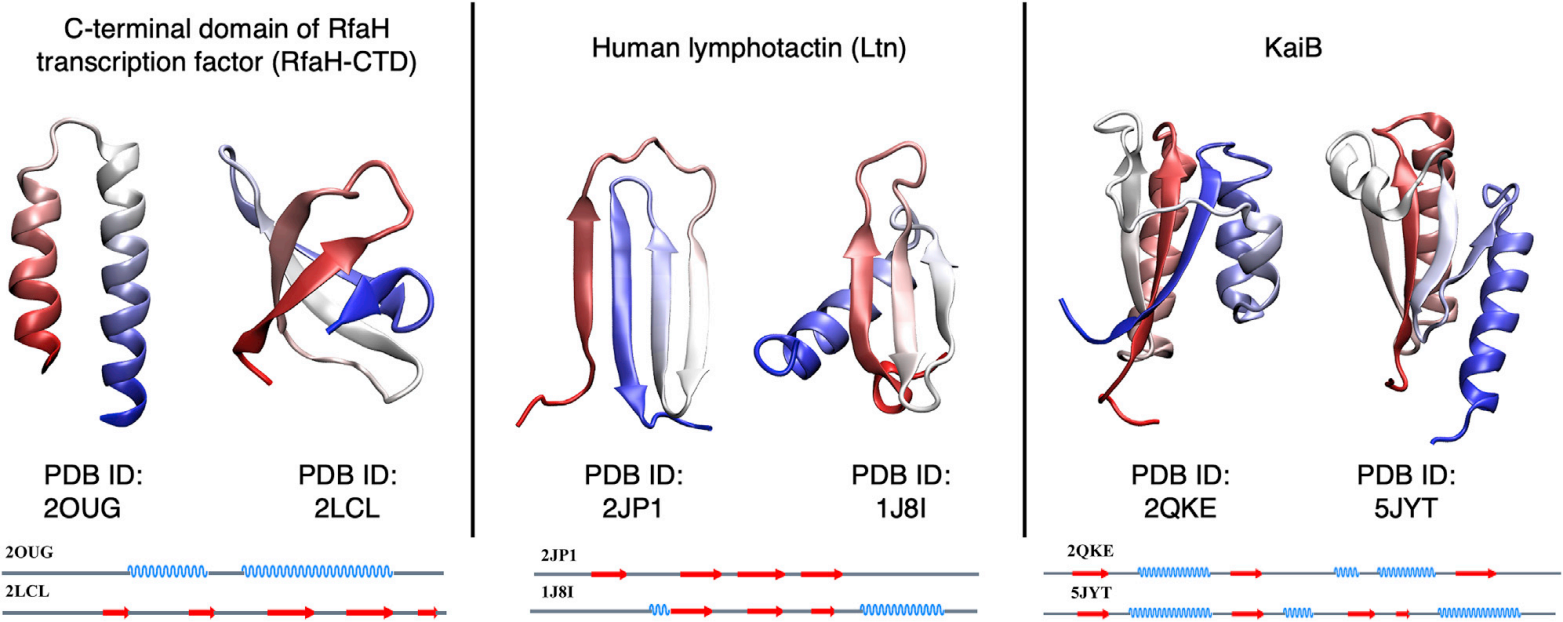
Representative examples of metamorphic proteins with three-dimensional structures of both folds. The protein backbone is colored from N-terminal (*red*) to C-terminal (*blue*). Secondary structure diagrams corresponding to the 3D structures are shown at the bottom of each panel. To see this figure in color, go online.

Christian Anfinsen was awarded a Nobel Prize in Chemistry in 1972 for his work on the apparent one-to-one relationship between the amino acid sequence of a protein and its three-dimensional fold (1,2), giving rise to the classic paradigm: “one sequence, one fold.” However, serendipitous discoveries in the past few decades have led to the identification of “metamorphic proteins” (3,4) that have the ability to jump reversibly between two distinctly different folds under native conditions. These proteins are fundamentally different from intrinsically disordered proteins (5), morpheeins (6), and moonlighting proteins (7,8), which have been studied for a long time. Typical conformational changes in proteins often involve “shearing” or “hinge” behavior in which entire protein subunits or secondary structure elements undergo relative motions without significantly altering the fold of the protein (9,10). In contrast, the different folds/structures of a metamorphic protein are dissimilar on a more fundamental level, often involving changes such as the transformation of a whole *α*-helix into *β*-strands (Fig. 1). In this article, we use significant change in secondary structure as the key defining characteristic of metamorphic proteins.

Although the number of known examples of metamorphic proteins such as IscU (11), RfaH (12,13), Selecase (14), Mad2 (15,16), XCL1 (also called lymphotactin) (17), CLIC1 (18), and KaiB (19,20) is relatively small, it is anticipated to increase steadily and populate the “metamorphome.” In all these metamorphs, the transition from one fold to another takes place in response to environmental triggers like pH, temperature, salt concentrations, binding partners, redox state, or oligomerization. Uncovering the metamorphome is crucial as it is expected to have a transformative effect on long-held concepts of protein structure and function. It could also lead to engineering of metamorphic proteins, which are molecular switches, to act as sensors of small molecules or local environmental changes.

Traditional x-ray crystallography techniques, which account for solving 90% of the protein structures in the Protein Databank (PDB), are limited in their ability to identify metamorphism in proteins. Although these methods did identify several metamorphic proteins, they trap the protein in a minimum free energy structure in a specific crystallographic environment, and thus, they usually do not reveal the existence of alternate folds if the protein is metamorphic. A powerful method to detect protein metamorphism is solution-state NMR. However, high-throughput screening protein sequences for potential metamorphic behavior by NMR is not feasible. A realistic approach would be to identify metamorphic candidates using computational approaches, which would allow experimental verification to focus on a smaller set of candidate proteins.

A recent computational study from Porter and Looger (21) identified 96-fold switching candidates in the PDB. The study stated that two characteristics of metamorphic proteins include discrepancies between experimentally derived and computationally predicted secondary structures and the occurrence of multiple independent subdomains that each fold cooperatively. Using these two metrics, they estimated that up to 4% of the proteins in the PDB may be metamorphic, which suggests that this class of proteins appears to be more common than those identified so far.

In this work, we propose a novel, to our knowledge, binary classifier for predicting protein metamorphism based on the diversity index, which takes advantage of the uncertainty in secondary structure prediction (SSP) methods. This method has a unique advantage in that it can predict metamorphic behavior in a protein of interest purely based on the amino acid sequence, without requiring a priori experimental knowledge of the three-dimensional structure. The classification method is trained using two reference data sets consisting of ∼200 manually annotated monomorphic and metamorphic sequences, respectively. We found a robust performance of the diversity index-based classifier with a Matthews correlation coefficient (MCC) of 0.355 (corresponding to ∼70% accuracy) that is largely insensitive to changes in the parameterization and training data set.

The rest of this article is organized as follows. We first give a brief overview of SSP methods as they provide the essential inputs into our classifier. Next, we introduce the diversity index (DI), which measures the uncertainty of predicted secondary structure, and we outline how the DI is used to classify a protein sequence as metamorphic or monomorphic. This is followed by a description of the reference data sets containing known metamorphic and monomorphic sequences used to train our classifier. The performance of the classifier is discussed in detail using metrics, such as the MCC, true positive rates (TPRs), and true negative rates (TNRs), and its robustness is tested using randomized cross-validation, sensitivity analysis, and examining how performance varies with different input SSP programs. We include a discussion of “outlier” protein sequences that are consistently misclassified by the DI-based model as well as how the performance depends on the sequence database for position-specific scoring matrix (PSSM) generation, an important auxiliary input for the SSP programs. The article concludes with some promising future directions.

## Methods

### Theory

#### SSP

Secondary structure (SS) is a property of amino acid residues within a protein structure that describes its local intrachain three-dimensional structure. Under the well-known DSSP system (22), secondary structure may be classified into eight states, which can be further reduced down to three: *α*-helix (H), *β*-strand (E), and random coil (C). In the years since the introduction of SS classification for known protein structures, several data-driven computational methods have emerged for SSP using only primary structure information, i.e., the amino acid sequence. Today, SSP is a vital part of the modern toolkit for protein structure prediction and design.

SSP methods can be understood in the conceptual framework of machine learning. The protein sequence is first processed into a feature vector consisting of information with structural relevance. Such features may include a PSSM, which estimates the probability distribution of amino acid residues at each position in the sequence, and is computed by performing sequence alignments to a sequence database (23) using programs such as PSI-BLAST (24). The feature vector is input into a neural network model, which has significant flexibility in its internal architecture, and provides three outputs for each amino acid representing the probabilities of *α*-helix (H), *β*-strand (E), and random coil (C), adding up to one. The parameters of the neural network are trained to reproduce known secondary structures from widely available structural data sets. The accuracy of three-state SSP for modern methods has been reported to be as high as 82–84% (25).

In this article, four widely used SSP programs were applied to predict the secondary structure of every sequence in our data sets, namely Psipred (26), SPIDER2 (27), SPIDER3 (28), and Porter 5.0, denoted here as Porter5 (29). Psipred, developed in 1999, introduced the idea of using the PSSM generated by PSI-BLAST as input to a feed-forward neural network for SSP. SPIDER2 uses a deep neural network that incorporates the PSSM from PSI-BLAST along with amino acid physicochemical properties (30) to predict secondary structures and main chain dihedral angles. SPIDER3 is an updated version of SPIDER2 that incorporates hidden Markov model sequence profiles generated by the HHBlits program (31) as input to a bidirectional recurrent neural network architecture, effectively allowing the entire sequence to calculate SS prediction at each position instead of a sliding window as in SPIDER2. Porter5 is the latest version of a series of SSP programs and uses HHBlits-generated HMM sequence profiles and PSI-BLAST-generated PSSMs as input. In this article, we used the UniProt90_2019_01 sequence database as the input to PSI-BLAST for PSSM generation, and the Uniclust30_2018_08 database was used as input for HHBlits. These published sequence alignment databases are distinct from the metamorphic and monomorphic reference data sets that were compiled as part of this work.

#### Metamorphic proteins and diversity index

Metamorphic proteins can reversibly adopt multiple folded conformations for the same amino acid sequence under native conditions (3,4). Moreover, representative examples of metamorphic proteins are characterized by significant differences in secondary structure between folds (Fig. 1), which is a distinct feature from more typical kinds of conformational change that generally preserve secondary structure, as described in the Introduction. Because these metamorphic proteins possess multiple stable folds with differences in secondary structure, our central hypothesis is that metamorphic protein sequences are able to “confuse” SSP programs. According to this hypothesis, we defined one descriptor, the diversity index (DI):

(1) $$DI={\left(P{\left(E\right)}^{2}+P{\left(H\right)}^{2}+P{\left(C\right)}^{2}\right)}^{-1},$$

where *P(E)*, *P(H)*, and *P(C)* are output quantities from the SSP program representing the probabilities of *α*-helix, *β*-strand, and coil, respectively, for a single residue in the sequence. The DI for a residue is the reciprocal of the well-known Herfindahl and Simpson indices (32) for quantifying diversity in a probability distribution, and its value ranges from 1.0 (100% probability of one output, 0% for the other two) to 3.0 (equal probability of all three outputs). The value of the DI is also equivalent to the exponentiated Shannon entropy (33) in the above limiting cases but takes on slightly different values for other distributions. High values of the DI indicate greater uncertainty in SSP. Because metamorphic proteins tend to have contiguous portions of the sequence (or even the whole protein) capable of undergoing changes in secondary structure, we also hypothesized that the DI of metamorphic protein sequences are elevated in contiguous regions of the sequence. Therefore, we consider the maximum value of a moving average of the DI over the sequence as the main criterion to predict metamorphic behavior in a protein sequence. In other words, a sequence will be classified as metamorphic if the following criterion is satisfied:

(2) $$\text{max}{\left\{\frac{1}{CR}\sum_{j=0}^{j<CR}DI_{i+j}\right\}}_{i=1}^{L-CR+1}>DI_{thre},$$

where the *CR* “number of consecutive residues” and *DI*_*thre*_ “diversity index threshold” are adjustable parameters. This binary classifier needs to be trained on reference or “manually annotated” data sets consisting of known metamorphic and known monomorphic (i.e., onefold) sequences. We will describe the construction of these data sets in the following sections.

### Data Set Setup

#### Construction of the metamorphic reference data set

In 2018, Porter and Looger published an article listing 192 metamorphic protein structures (96 pairs) (21) in which most pairs have very high sequence similarity to one another (between 90 and 100%). In eight cases, both metamorphic protein structures were found as different chains with identical sequences in a single deposited assembly (e.g., PDB ID: 5C1V). Our metamorphic reference data set, listed in Table S1, makes the following revisions to the listing in (21). Among the original set of 192 structures, one protein is no longer available from the database (2A01). We also removed proteins in which the fold switching region is contained within 20 residues of the N- and C-termini (4ZRB, containing two structures) or if the sequence length is shorter than 40 residues (4FU4, 4G0D, 5K5G, and 2KB8); this is because our classifier requires taking a moving average of the diversity index, requiring a sequence that is longer than the largest window size (>15 residues) plus the number of the removed terminal residues (>5 × 2 residues). In total, eight proteins were removed from the list for the reasons above. We also added several proteins to the list, including the designed sequence pair G_A_/G_B_ from protein G (34) that was excluded from (21) (PDB ID: 2LHC and 2LHD) and 15 other possible metamorphic proteins that have experimental evidence for metamorphism but one solved structure, such as 2LSH. Therefore, our reference metamorphic data set contains 192 − 8 + 15 + 2 = 201 metamorphic protein structures in total.

#### Construction of the monomorphic reference data set

Our classification model for predicting protein metamorphism needs to be trained on proteins with known metamorphic behavior, as well as those with known single-fold (i.e., monomorphic) behavior. Although it is widely assumed that the PDB contains mostly monomorphic proteins, it is likely that a significant portion exhibits as yet undiscovered metamorphic behavior. Therefore, we queried the PDB to obtain a set of protein structures that are highly likely to be monomorphic based on the following set of filtering criteria, which were adjusted to produce a data set with a similar size as the metamorphic set: 1) the structure should be reported at least 10 years ago and has a good quality structure in the sense that x-ray structures with resolution >2.2 Å were filtered out; 2) there must be >30 published structures with at least 50% sequence similarity with the structure of interest; and 3) the sequence length is >40 and <250 residues to meet the criteria of having a well-folded core while staying within the typical sequence lengths of globular proteins. Each structure found in the above manner is termed “parent protein,” and structures with high sequence similarity found in step (2) above are termed “child proteins.”

A total of 1387 “parent protein” structures with a maximum sequence similarity of 70% and more than 65,000 “child protein” structures were downloaded along with their abstracts from the Research Collaboratory for Structural Bioinformatics Protein Databank (RCSB PDB) web server using an automated crawler written in Python that uses the *scrapy* package. Two filtering rules were imposed to maximize the probability that a “parent protein” is monomorphic:

1) The root mean-square deviation (RMSD) values were calculated for all pairs of structures after sequence alignment for a “parent protein” and all of its “children.” The structure was excluded from the data set if any of the pairwise RMSD values exceeded 2.4 Å.
2) The mismatch in secondary structure (SS) was calculated for all pairs of structures after sequence alignment with a “parent protein” and all of its “children.” A positional mismatch score is calculated by summation over aligned residues in a window of 30 residues in length, where “2” was assigned if one sequence is H and the other is E, and “1” was assigned if one sequence is C and the other is either E or H, and then taking the maximum value over all window positions. The structure was excluded from the data set if the SS mismatch score between any pair of sequences exceeded 9.

Finally, the abstracts of the corresponding publications were checked for keywords such as “fold switching,” “metamorphic,” “two folds,” and other synonyms; if the abstract indicated possible metamorphic behavior, then it was excluded from this data set as well. This procedure resulted in a total of 136 likely monomorphic proteins (Table S2).

**Figure 2:**
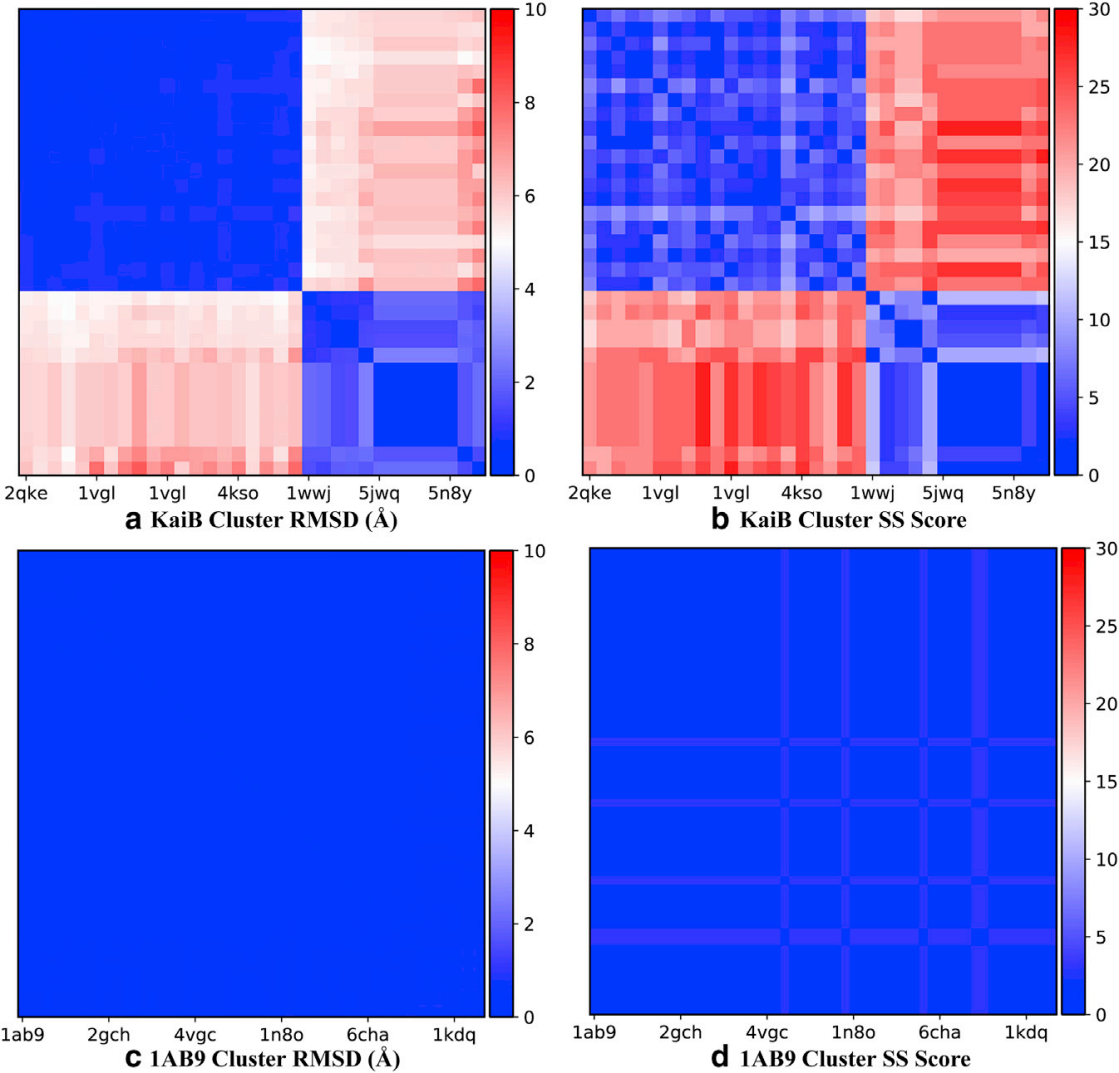
RMSD (*a* and *c*) and SS (*b* and *d*) score of KaiB and 1AB9, respectively. The highest RMSD value and highest SS score of the KaiB cluster is much larger than those of the 1AB9 cluster. To see this figure in color, go online.

An example of a metamorphic protein (KaiB) and a monomorphic protein (1AB9) from our reference data sets is shown in Fig. 2. The highest RMSD value in the KaiB (typical example of metamorphic proteins) cluster exceeds 7.0 Å, and many pairs of sequences exhibit a secondary structure mismatch of 23 or greater. On the contrary, both the RMSD values and SS score are consistently low for the monomorphic protein 1AB9.

## Results and Discussion

### Behavior of the diversity index (DI)

**Figure 3:**
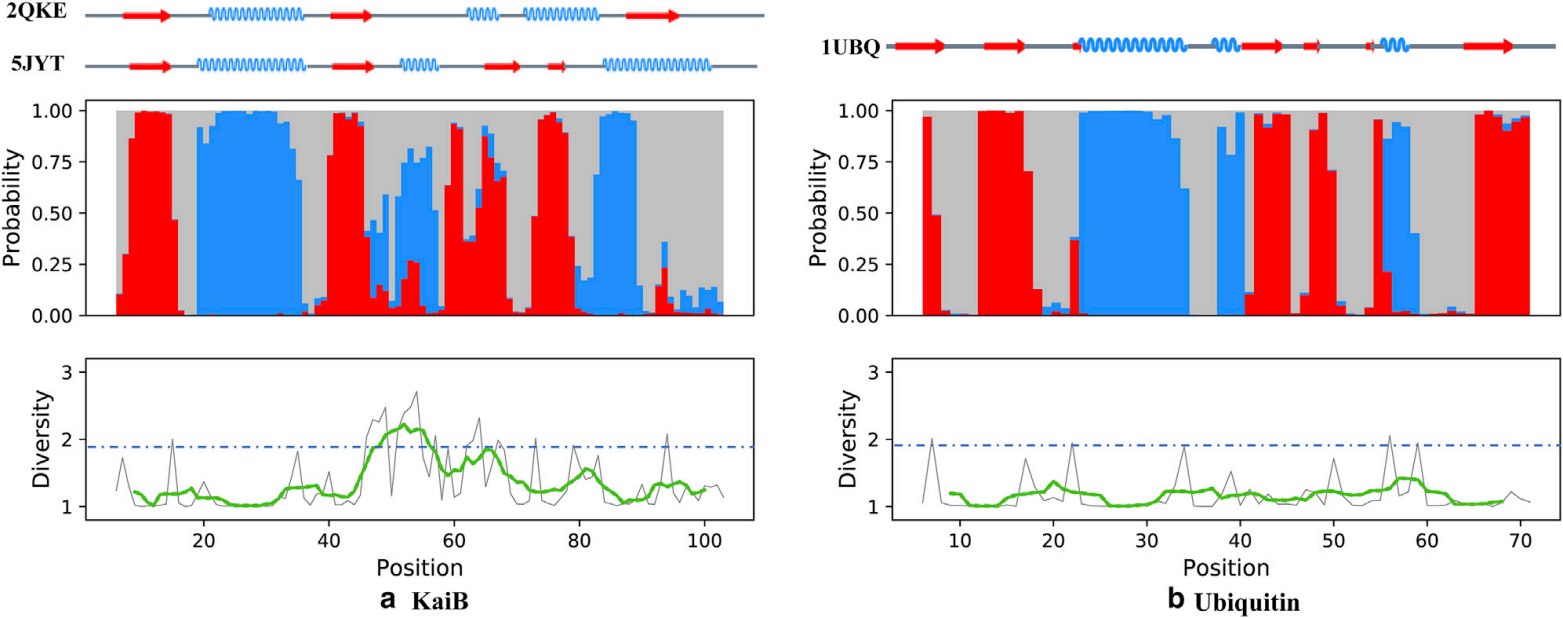
SSP results for KaiB (*a*) and ubiquitin (*b*). Top: experimentally derived SS of both KaiB structures (PDB ID: 2QKE and 5JYT, *left*) and ubiquitin (1UBQ, *right*). Middle panel: stacked bar plots show predicted SS probabilities at each position in the sequence from SPIDER2 (*red*, strand (E); *blue*, helix (H); and *gray*, C). Bottom panel: diversity indices for each residue position in the sequence (*gray*), with a moving average, window size of 14 (*green*), and DI threshold, *DI_thre_*, for metamorphic behavior (*blue dotted line*). The DI takes on higher values when the predicted SS is more evenly distributed between H, C, and E, indicating greater uncertainty. For KaiB, the higher DI regions coincide with the experimentally known metamorphic regions (19). To see this figure in color, go online.

According to Eq. 1, the range of the diversity index (DI) is from 1 to 3, with larger values indicating greater uncertainty of SS prediction. Fig. 3 plots the SS and DI from the SPIDER2 program for a well-known metamorphic sequence (KaiB, *left panel*) and monomorphic sequence (ubiquitin, *right panel*) along with the experimentally derived secondary structure(s). As shown in the left panel, the DI of the KaiB sequence has several regions of elevated values in the metamorphic region that spans positions 50–90. On the other hand, the DI of ubiquitin is relatively low for the whole sequence, with small jumps at the boundaries of different secondary structure domains that are smoothed out by taking the moving average. This example illustrates how diversity indices may be used to predict metamorphic behavior in proteins when the folds exhibit different secondary structure in the metamorphic regions.

### Diversity index-based classifier performance

The performance of our model is measured using the MCC, a well-established measure of the quality of binary classifications. For each combination of our parameters *CR* and *DI*_*thre*_, the MCC is computed from a matrix of true positives (TP), false positives (FP), true negatives (TN), and false negatives (FN), called a confusion matrix (35):

(3) $$MCC=\frac{TP\times TN-FP\times FN}{\sqrt{\left(TP+FP\right)\left(TP+FN\right)\left(TN+FP\right)\left(TN+FN\right)}}.$$

The value of the MCC ranges from −1 to +1, where random classification gives a value of 0, perfect classification gives +1, and “perfectly wrong” classification gives −1 (equivalent to perfect classification if all predictions are reversed). For context, a recent review of machine learning methods for predicting disease in individuals reports MCC values ranging from −0.24 to +0.55 (36). An advantage of MCC is that the positive and negative data sets play equally important roles, even if they are imbalanced in size. We also report simpler measures of TPR, TNR, and accuracy, defined as:

(4) $$TPR=\frac{TP}{TP+FN}\;\;\;\;\;\;\;\;\;\;TNR=\frac{TN}{TN+FP}\;\;\;\;\;\;\;ACC=\frac{TP+TN}{TP+TN+FP+FN},$$

which range from 0 to 1, and random classification gives 0.5. The accuracy is intuitive because it is simply the ratio of correct predictions to the total number of data points, but we do not use it in training because it can hide the effects of imbalanced performance for positive and negative cases.

**Figure 4:**
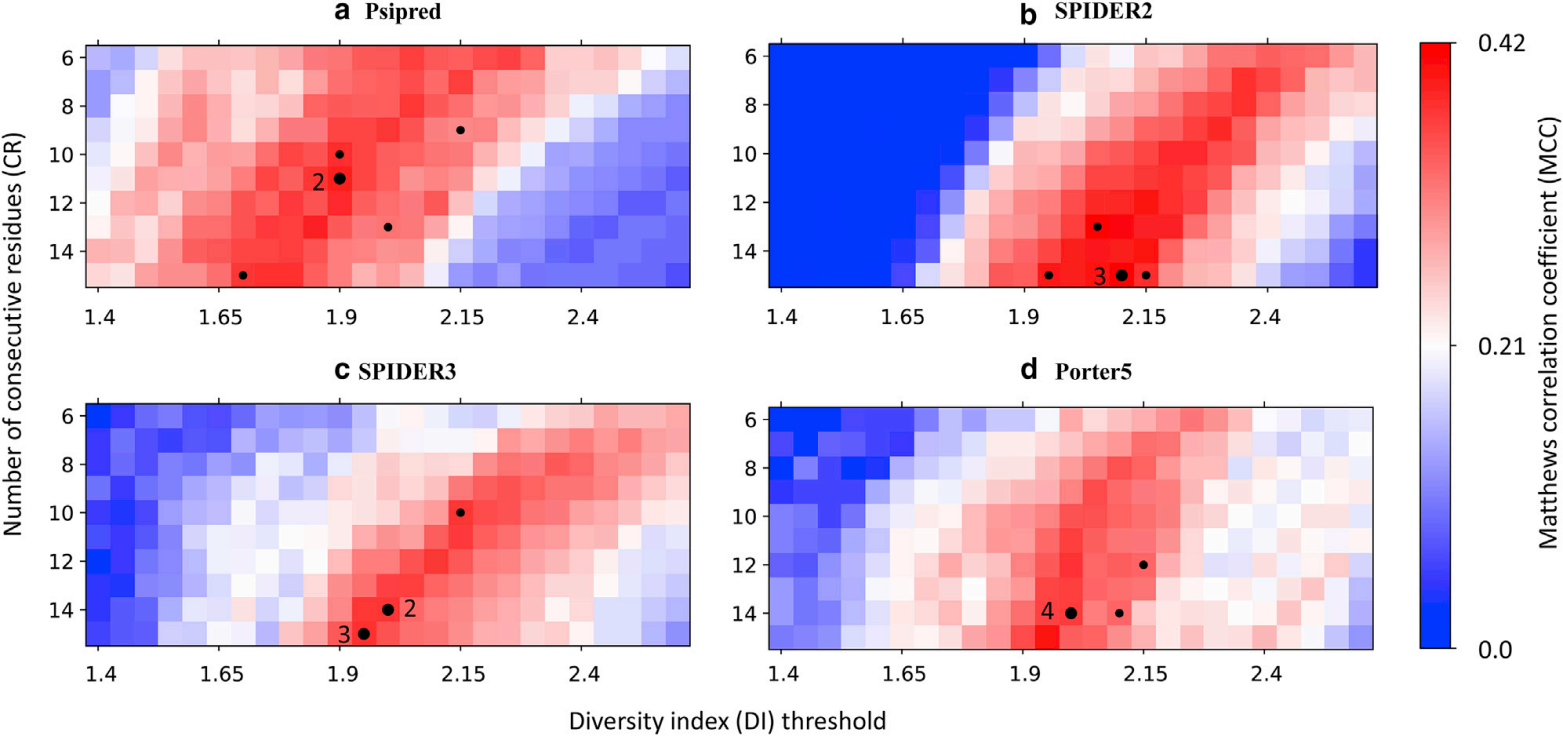
MCC heat maps for the diversity index-based classifier using predicted secondary structures from four programs, namely (*a*) Psipred, (*b*) SPIDER2, (*c*) SPIDER3, and (*d*) Porter5. The color map (*blue* < *white* < *red*) corresponds to MCC values computed for the full reference data set. Each black dot indicates the optimized parameter value for a randomly selected training set (∼84.3% of the full data set), with numbers indicating how many times each optimum was found. To see this figure in color, go online.

Generally speaking, larger values of *CR* correspond to increased window size and tend to decrease the maximum value of the moving average. Larger values of *DI*_*thre*_ also tend to decrease the probability that a sequence is classified as metamorphic. Thus, for increasing values of *CR* and *DI*_*thre*_, the true negative and false negative rates both increase. To get a better understanding into the behavior of our model, we plotted heat maps of the MCC in our two-dimensional parameter space shown in Fig. 4. Herein, we consider possible values of *CR* ranging from 6 to 15 and possible values of *DI*_*thre*_ ranging from 1.4 to 2.6 with a step size of 0.05.

The sensitivity of our model was tested by cross-validation. In general, cross-validation involves partitioning the data set into training set and test set and verifying the model obtained from the training set by making predictions for the test set. Here, we applied six-fold cross-validation. The complete data set including monomorphic and metamorphic proteins was randomly shuffled and split into six even-sized chunks. In each of the six trials, five selected chunks were treated as the training set and the remaining chunk as the test set. The parameters were determined by maximizing the MCC for the training set and then used to calculate the MCC for the test set.

Fig. 4 and Table 1 show the main results for our DI-based classification using four SSP programs. Similar levels of performance for the training set were obtained using all four SSP programs as input to the DI-based classification. Among these methods, SPIDER2 had the highest average MCC value of 0.418 for the training set (Table 1), which was slightly higher than that of Porter5 (0.393), SPIDER3 (0.401), and Psipred (0.311); the differences were rather small and within the standard errors from randomized cross-validation trials. The parameters that maximized the MCC tended to appear in the middle of the parameter space, with significant regions of the parameter space exhibiting only minor variations from the optimum. For example, in the case of SPIDER2, the largest MCC value among all the trials was around *CR* ∼15 and *DI*_*thre*_ ∼2.1.

**Table 1:** The MCC Results from Four Different SSP Programs, Including the Training Set Results and the Test Set Results

MCC	Psipred	SPIDER2	SPIDER3	Porter5
Training set	0.311 (0.018)	0.417 (0.015)	0.401 (0.017)	0.393 (0.035)
Test set	0.255 (0.091)	0.355 (0.104)	0.327 (0.124)	0.272 (0.096)

Numbers in parentheses are sample SDs over cross-validation trials.

In terms of the test set, Porter5 and Psipred performed similarly with MCC values of 0.25–0.28, with differences being within the standard errors from randomized cross-validation trials. SPIDER2 had the highest average MCC value (0.355) for the test set. The small difference between the test set and training set MCCs and the consistency of our results across several models indicate that the DI-based classifier is a robust method for predicting metamorphic behavior. SPIDER3 showed moderate performance in the test set compared with the other three methods, with a MCC of 0.332. Fig. 4 *b* also shows that SPIDER2 had a broader range of parameter space with near-optimal performance as compared to Psipred, SPIDER3, and Porter5. The training and test results overall indicate that higher SSP accuracy does not directly translate to better performance in metamorphic protein classification.

Although these methods had similar MCC values, their accuracy in terms of correctly predicting TP and TN showed much greater variations. According to the data shown in Table 2, the TPR is lower than the TNR in all four methods for the optimum parameters that maximized the MCC. Among these methods, SPIDER3 had by far the highest TNR value (0.92) and lowest TPR value (0.42). The other three methods had similar TPR ranging from 0.59 to 0.66 and TNR ranging from 0.78 to 0.83, which are within the limits of statistical errors from our cross-validation studies. We presumed that the large TNR values of SPIDER3 comes from overall low values of the calculated diversity index, which possibly originates from higher SS prediction confidence levels as compared to other methods. We thus recommend SPIDER2 as the input method of choice for metamorphic protein classification because of its consistently high MCC value for both training and test sets, balanced TPR and TNR, and wide regions of parameter space with near-optimal performance.

**Table 2:** TPR, TNR, and Accuracy of Test Set for Four Different SSP Programs

Measure	Psipred	SPIDER2	SPIDER3	Porter5
TPR	0.598 (0.113)	0.633 (0.087)	0.411 (0.017)	0.581 (0.018)
TNR	0.707 (0.071)	0.782 (0.064)	0.918 (0.023)	0.763 (0.062)
Accuracy	0.649 (0.021)	0.698 (0.015)	0.633 (0.016)	0.645 (0.027)

Numbers in parentheses are sample SDs over cross-validation trials.

### Comparison with other methods

There currently exist a few methods in the literature for predicting metamorphic behavior in proteins (21,33). To our knowledge, all existing methods require the knowledge of either the protein’s empirical three-dimensional structure or secondary structure. Porter et al. hypothesized that metamorphic proteins possess at least one domain with a fold that is largely independent of the rest of the sequence and proposed a method to predict metamorphic behavior based on the prediction of independent folding domains (21,37). This method requires the protein’s experimentally determined three-dimensional structure, and thus, its predictions are based on existing structural knowledge.

More recently, Porter et al. and co-workers reported that metamorphic proteins have lower SSP accuracy than monomorphic proteins or fragments (38), which is similar to the ideas in our current work; however, the method they proposed requires prior knowledge of experimental secondary structure. A major differentiating feature of the diversity index-based classification method presented here is that it requires no experimental data for the sequence of interest. Thus, this method could be used to make predictions of metamorphism in protein sequences in which there is no existing structural data.

### Classification using multiple diversity indices

**Figure 5:**
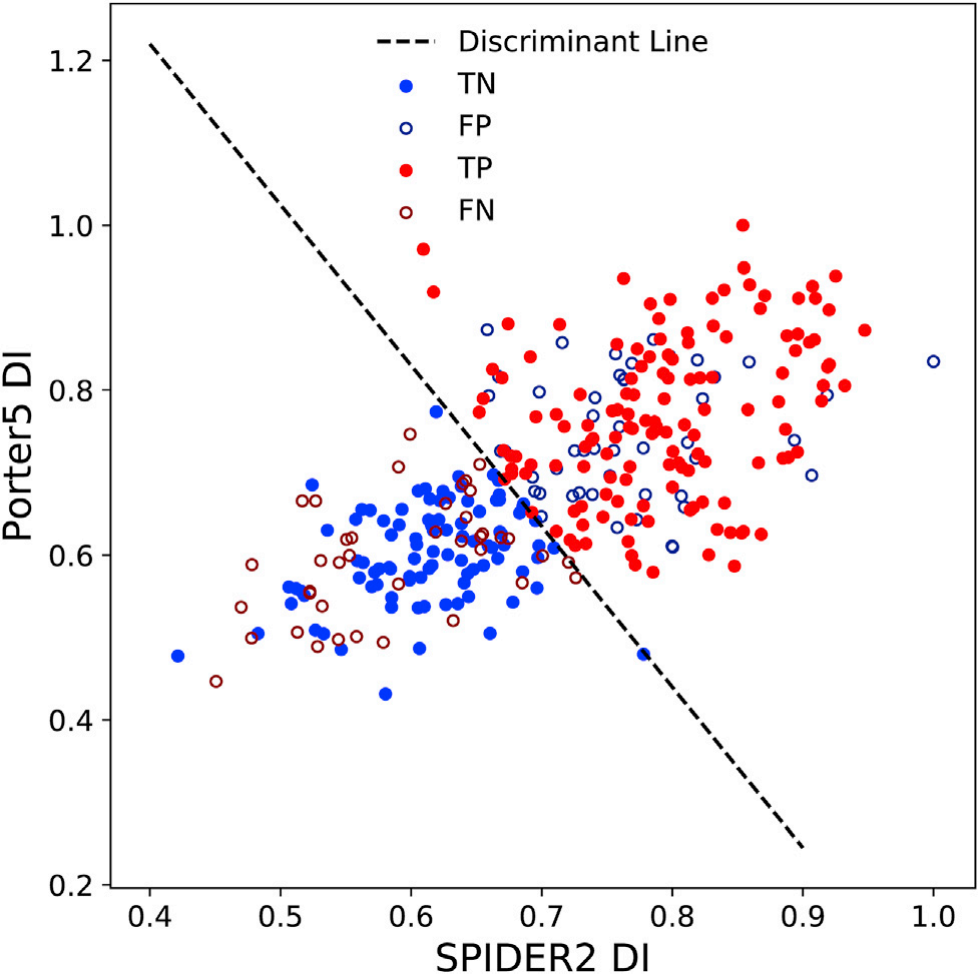
Two-parameter classification using diversity indices from SPIDER2 and Porter5. Here, the TP (metamorphic proteins in the reference data set correctly classified as metamorphic) are represented by red filled circles, and the false negatives (metamorphic proteins in the reference data set incorrectly classified as monomorphic) are represented by red open circles. The blue filled circles and blue open circles represent TN (monomorphic proteins in the reference data set correctly classified as monomorphic) and false positives (monomorphic proteins in the reference data set incorrectly classified as metamorphic), respectively. To see this figure in color, go online.

We also examined the possibility of obtaining an improved classification model based on a linear combination of DIs obtained from two SSP programs, essentially increasing the number of descriptors to two. The discriminant parameters (i.e., slope and intercept of the line) were optimized by maximizing the MCC (35). Using a linear combination of the SPIDER2 DI and the Porter5 DI, we found the MCC value of the optimal model increases to 0.45. Fig. 5 plots the discriminant line and the descriptor values for each protein as a scatter plot. The diagonal shape of the distribution indicates a high degree of correlation between the two diversity indices (R^2^ = 0.41), and most of the metamorphic proteins identified as TP are located in the top-right corner of the figure. We found a similar performance using some alternate approaches, for example an “inconsistency index” to predict metamorphism using the level of disagreement between two SSP programs (Fig. S1; (39)) and principal component analysis on the results of multiple SSP programs followed by K-means clustering (Fig. S2). These methods all yielded results with MCC values within 0.1 of the basic method using a single diversity index.

However, our analysis also revealed some false negatives (FN, *open red circles*) in the lower left of Fig. 5; these are metamorphic proteins in our reference data set but have very low diversity indices and contradict our rationale for the DI-based classifier. The same applies for false positives (FP, *open blue circles*) in the upper right of the Fig. 5 as these are monomorphic proteins in the reference data set with high diversity indices. In the following section, we provide a rationale to explain these outliers.

### Analysis of outliers in diversity index-based classification

Several proteins are consistently misclassified by the DI method using the predicted SS from all four programs. There are 22 metamorphic proteins in our reference data set that are consistently misclassified as monomorphic proteins (false negatives) and 14 monomorphic proteins consistently misclassified as metamorphic proteins (false positives).

We examined the 22 “persistent” false negatives, i.e., metamorphic proteins from our reference data set that are consistently misclassified as monomorphic, and generally found that their two folds did not satisfy our initial criterion of having significantly different secondary structures and instead feature other kinds of conformational differences, which we discuss in the following examples. The three-dimensional structures of the false negatives are shown in Fig. S3.

1) 2LQW (40) and 2BZY (41): A closer examination reveals that these two structures have very similar secondary structures in spite of different folds. As shown in Fig. S3, 2LQW is a key signaling protein that exists as a monomer, whereas 2BZY is a partial structure of a CrkL homodimer protein, in which the existing part has similar SS to 2LQW. Moreover, the truncated CrkL monomer protein (PDB ID: 2BZX) has a highly similar secondary structure to 2BZY. The high similarity in secondary structure is consistent with the classification assigned by our method, which is based on differences in secondary structure between folds.
2) 2NNT (42) and 2MWF (43): 2NNT is a tetrameric amyloid protofilament that forms an extended *β*-sheet between multiple chains, whereas 2MWF is a mutant monomer that forms a *β*-sheet within the residues in one chain. Again, the highly similar secondary structure in both folds is consistent with the classification assigned by our method.
3) 4HDD (44) and 2LEP (45): The structures in this pair are similar in terms of secondary structure but have a large RMSD. 4HDD is a homodimer in which a *β*-sheet is formed between chains, whereas 2LEP uses the same domain to form a *β*-sheet within one chain.
4) 1G2C (46) is a truncated protein whose SS closely matches with the corresponding residues in 5C6B, which is a full structure. Strikingly, the other protein 5C6B (40), which has a similar sequence to 1G2C, is correctly classified by SPIDER2 and Porter5 as a metamorphic protein. The high DI domain of 5C6B (residue 270–295) was not part of the 1G2C structure, which indicates the incorrect classification of 1G2C might be solely because of the truncation of the input sequence.
5) 4XWS (47) and 4Y0M (48): Upon examination of the structures, we think this structure pair had been incorrectly included in our reference metamorphic data set as these two structures are highly similar in terms of secondary structure as well as three-dimensional structure (RMSD: 1.561 Å). In fact, the text of (47) states that the metamorphic region of the protein could not be solved by x-ray crystallography.

Within the 14 persistent false positives, i.e., monomorphic proteins from our reference data set that are consistently classified as metamorphic, we found the following examples, with 3D structures shown in Fig. S4:

1) 2UU8 (49) is a concanavalin A protein, and its DI value is relatively high in all the SSP programs, particularly in SPIDER2 (∼2.5). This structure possesses many short adjacent domains with different secondary structures, which leads to uncertainty in the SSP programs. Also, *β*-sheets are dominant in the SS of 2UU8, and we observed that the outermost strand of the *β*-sheet has a general tendency to have high uncertainty from SSP programs.
2) 3SEB (50) is a protein with several short SS domains (including *α*-helices and *β*-sheets), leading to the high DI values for these domains, similar to the example above. More than half of the false positives follow the same trend, indicating that our DI-based classifier is biased to misclassify protein sequences that are monomorphic but intrinsically difficult for SSP programs because of having many short subdomains with distinct SS or outermost *β*-strand among several (anti-)parallel *β*-sheet strands.
3) 2JE7 (51), a recombinant protein made from the seed lectins of two *Dioclea* species, has the same situation that the outermost strand of *β*-sheets, and the short adjacent SS segments have the highest DI values. However, this protein is known to form either a dimer or a tetramer depending on the pH value, and environmental-mediated changes in stoichiometry are a known driving force of protein metamorphism. Although there exists no direct evidence of SS change in this dimer-tetramer equilibrium process, it is possible that this process is associated with metamorphism not yet discovered.
4) 3CHB is another monomorphic protein whose DIs are very large in all the SSP programs. Unlike the other two false-positive proteins above, each subunit of the homopentameric 3CHB (52) has a long *α*-helix and six medium-length *β*-strands. Another short-length *α*-helix is located at the N-terminus. According to the SPIDER2 prediction, three out of six *β*-strands have relatively large DIs, resulting in a region with a high average DI value. So far, we do not have a good explanation for the reason of this false positive. One possibility is that other proteins in the PDB have highly similar sequences to these high DI *β*-strands but have different SS.

We note that it is possible for our reference monomorphic data set to include proteins that are actually metamorphic, despite our efforts to minimize this occurrence. This is because our selection of monomorphic proteins was based on the analysis of known structures in the PDB, which is missing alternate folds or structures that have not yet been discovered or deposited.

### Dependence of results on sequence database

**Figure 6:**
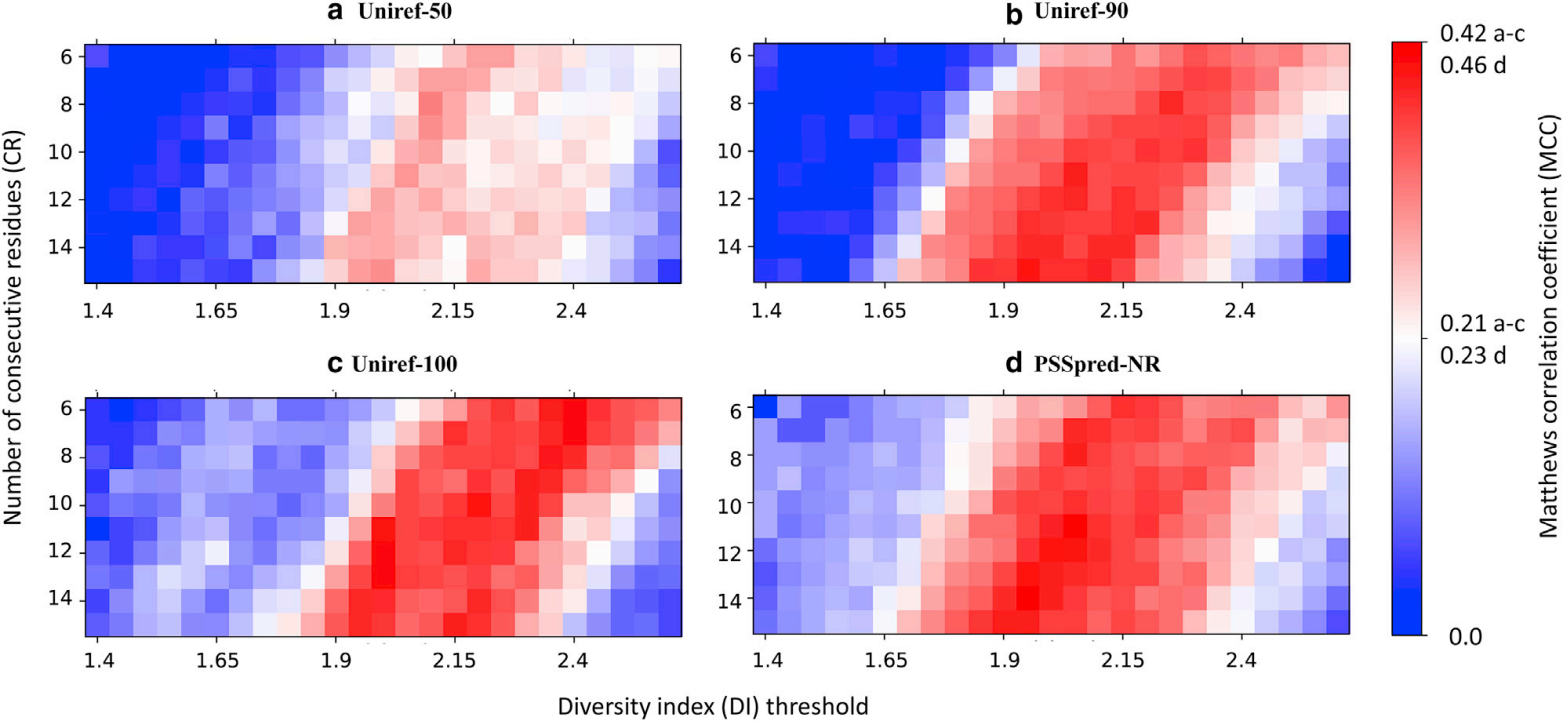
The different MCC values calculated based on (*a*) 50% nonredundant sequence data set, (*b*) 90% nonredundant sequence data set, (*c*) 100% nonredundant sequence data set, and (*d*) PSSpred nonredundant sequence data set. To see this figure in color, go online.

The performance of SSP programs relies on the nonredundant sequence database that is used to compute the PSSM. Fig. 6 shows the differences in classification performance when SPIDER2 is used as the SSP program for different choices of the nonredundant sequence database. The Uniref-50, Uniref-90, and Uniref-100 databases have progressively larger numbers of sequences and sequence identities among pairs of sequences. Fig. 6 shows that Uniref-50 has a markedly lower performance for our classifier compared to Uniref-90 and Uniref-100, and it is currently unclear whether the poor classification performance is due to the smaller size of the sequence data set or the more stringent threshold on sequence identity. Surprisingly, the modified nonredundant sequence data set (53) from I-TASSER (PSSpred) gives a very high MCC value (0.457), even though it was released in 2014 and has not been continually updated like the other three. Thus, the DI-based classification performance depends on the sequence database in a nontrivial way and does not necessarily yield improved results for updated database versions.

## Conclusions

In this article, we described a diversity index-based classification model to predict metamorphic behavior in proteins solely based on the protein sequence. Our model was trained on a reference data set consisting of 136 known monomorphic proteins and 201 known metamorphic proteins. Although the main purpose of SSP programs is to predict secondary structure, our results indicate that the “byproducts” of SSP, namely the alternate SS probabilities and the derived diversity index, can play a key role for predicting metamorphism in proteins. Among the four popular SSP programs, SPIDER2 has the overall best performance and robustness in classifying proteins as monomorphic versus metamorphic. Further improvements in performance may be obtained by comparing the output of multiple SSP programs. Because all four SSP programs give similar MCC values when used in classification to within ∼10%, we think further improvements in predicting protein metamorphism will require SSP methods that focus more on accurate quantification of uncertainty rather than yielding the best fit to experimental data. There is also potential for improvement in curating the annotated metamorphic and likely monomorphic data sets; for example, the thermodynamic stability of the native state could be used as a criterion for a likely monomorphic protein (37).

Our examination of false positives and false negatives illustrates both the predictive potential and the limitations of the DI-based approach. In terms of false positives, we found some indications of undiscovered metamorphic behavior in the monomorphic data set, possibly driven by pH-dependent changes in stoichiometry. On the other hand, the false negatives highlight fold switching behavior in proteins that is not well described by significant changes in secondary structure. This indicates that metrics going beyond SSP may be needed to predict certain kinds of protein metamorphism, which is a promising direction of future research.

## Author Contributions

N.C. conceived the presented idea and carried out the research. N.C., A.L., and L.-P.W. designed the research. N.C. and L.-P.W. wrote the manuscript. M.D. and A.L. provided important feedback on the research and manuscript, including ideas for data analysis and the broader context of the research. All authors provided feedback and revisions on the manuscript.

## Supporting Material

Document S1. Supporting Materials and Methods, Figs. S1–S4, and Tables S1–S2

Document S2. Article plus Supporting Material
