# Supplementary material for: Sequence-Based Prediction of Metamorphic Behavior in Proteins
Source: Biophys J. 2020 Aug 14;119(7):1380–90. doi: 10.1016/j.bpj.2020.07.034 (PMC7567988; doi:10.1016/j.bpj.2020.07.034)
Supplement: Document S1. Supporting Materials and Methods, Figs. S1–S4, and Tables S1–S2 [file mmc1.pdf]

Biophysical Journal, Volume *119*

Supplemental Information

Sequence-Based Prediction of Metamorphic Behavior in Proteins

Nanhao Chen, Madhurima Das, Andy LiWang, and Lee-Ping Wang

Inconsistency Index. Besides the diversity index, there is another index describing the ‘confusion’ of the SSP programs, named the inconsistency index (iCI) because it evaluates the differences in predictions between two SSP programs. When a residue in a sequence has identical SSP results from two programs, then we assign an inconsistency value of 0 to that position. The assigned value is 1 when one prediction is random coil (C) and the other is either helix (H) or sheet (E). The assigned value is 3 when one prediction is helix (H) and the other is sheet (E). Similar to the DI, two variables are used to optimize the performance of the iCI, which are the number of consecutive residues in the moving window (CR, vertical axis) and the threshold value of the iCI (horizontal axis). The results are plotted as a heat map similar to Figure 4 in the main text. The SS comparison between Porter5 and Psipred has the MCC value as large as 0.3951, which is comparable to the MCC value obtained by the DI descriptor.

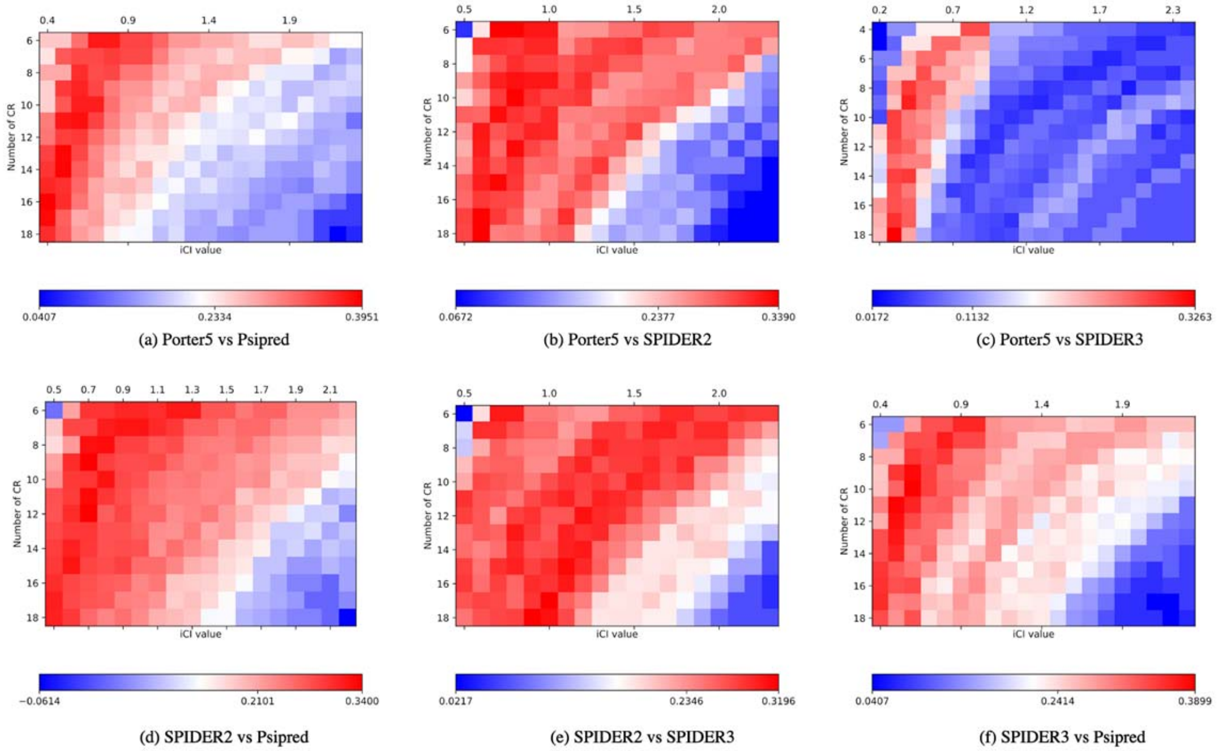

Supplementary Figure S1. The MCC map of the IC descriptor of six SSP programs comparison, including the SS comparison between (a) Porter5 and Psipred, (b) Porter5 and SPIDER2, (c) Porter5 and SPIDER3, (d) SPIDER2 and Psipred, (e) SPIDER2 and SPIDER3, and (f) SPIDER3 and Psipred.

Principal Component Analysis (PCA).

The unsupervised PCA method followed by K-means clustering was used to separate all the data into two groups, namely metamorphic (positive) and non-metamorphic groups (negative). After clustering, we calculate the MCC value as 0.41. This result indicates that without knowing the metamorphic property of proteins, the DIs are still appropriate for separating metamorphic proteins and non-metamorphic proteins.

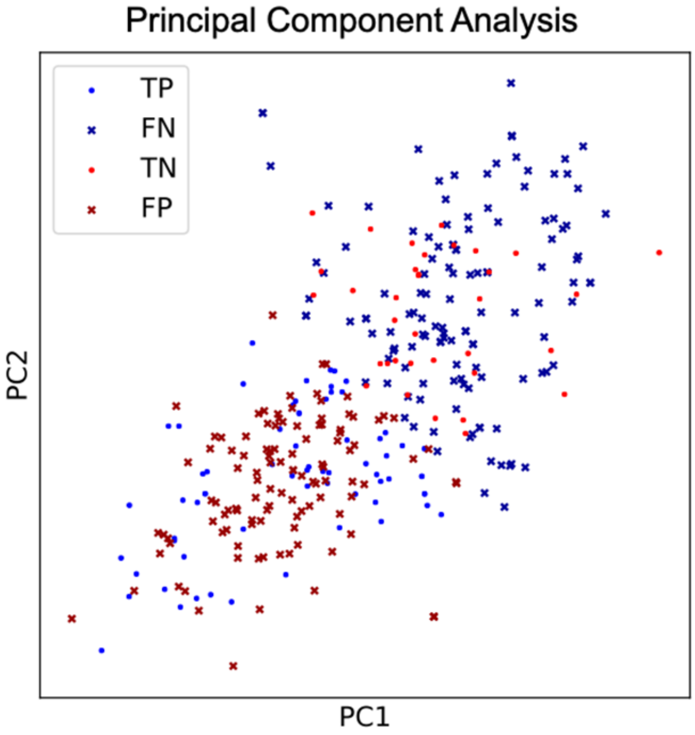

Supplementary Figure S2. The PCA result of two most important principal components.

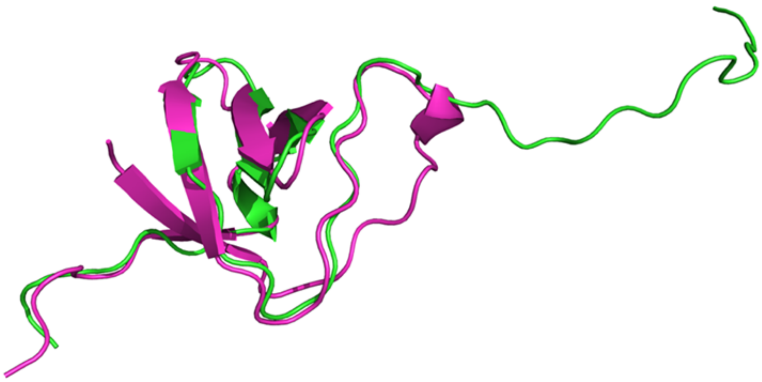

(a) 2LQW (magenta) and 2BZY (green).

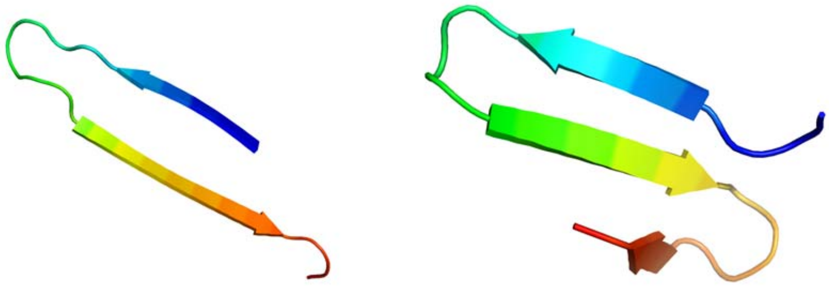

(b) 2NNT (left) and 2MWF (right), colored by residue number from blue (low) to red (high).

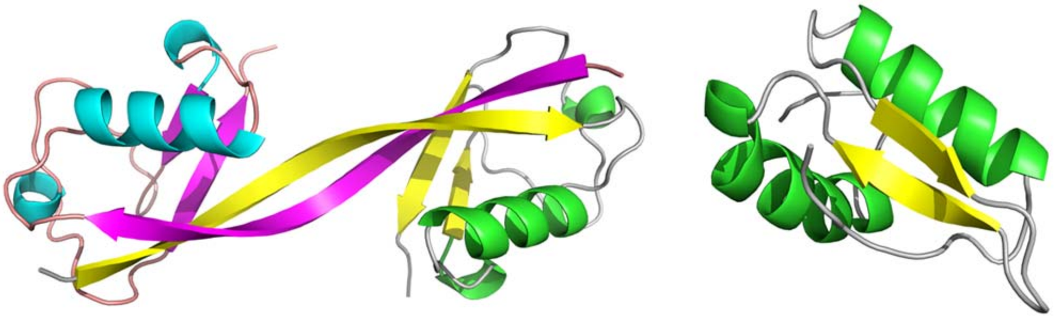

(c) 4HDD (left) and 2LEP (right). Green: first chain helix, yellow: first chain strand, cyan: second chain helix, magenta: second chain strand.

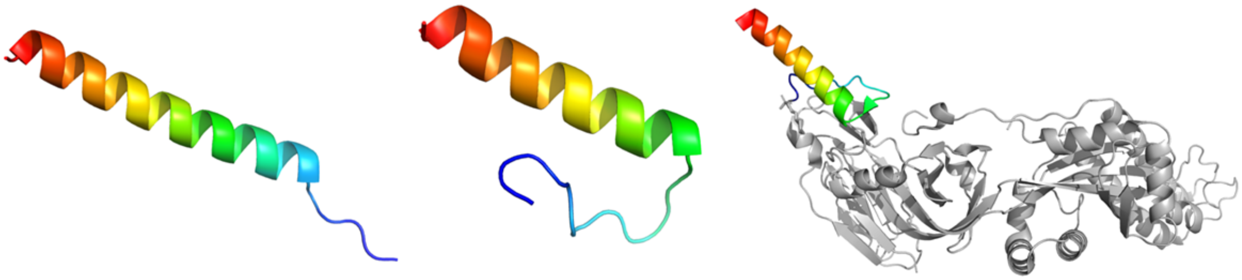

(d) 1G2C (left), truncated 5C6B (center) and entire 5C6B (right), colored by common range of residue numbers.

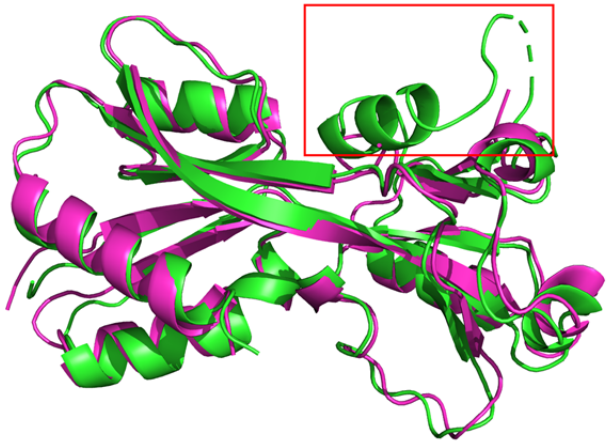

(e) 4XWS (magenta) and 4Y0M (green).

Supplementary Figure S3. Five of the persistent ‘false negative’ results for diversity index-based metamorphic protein classification. Upon closer examination of these structures, we think these proteins should have been annotated as monomorphic, or at least excluded from the metamorphic dataset (see main text for details).

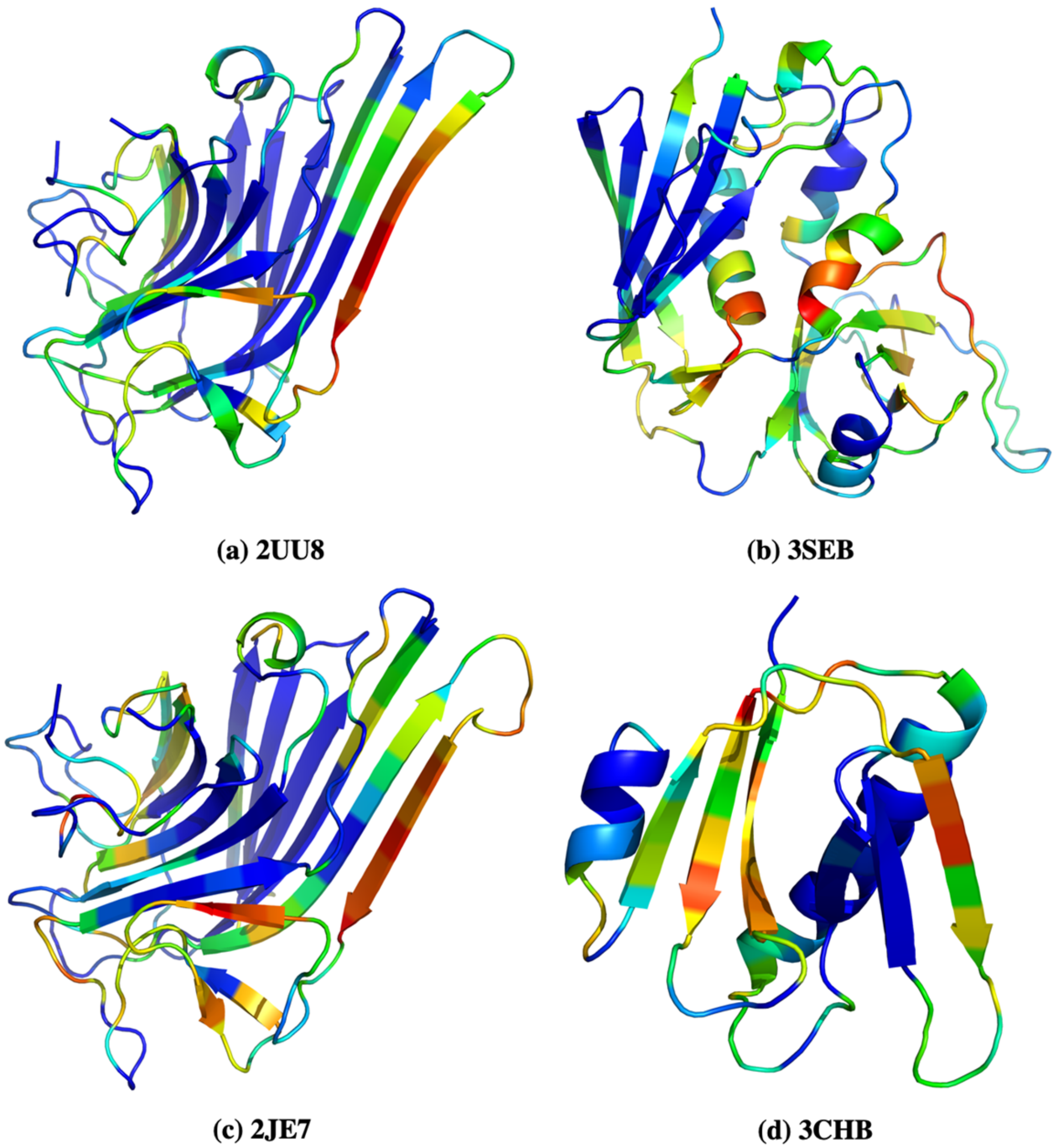

Supplementary Figure S4. Four of the persistent ‘false positive’ results for diversity index-based metamorphic protein classification: (a) 2UU8, (b) 3SEB, (c) 2JE7 and (d) 3SEB. The secondary structures of the proteins are colored based on diversity indices at each position, ranging from 1.0 (blue) to 3.0 (red).

Supplementary Table S1. The metamorphic dataset. Each row indicates one pair of proteins and the sequence similarity of the full protein sequence. Numbers in parentheses indicate the sequence similarity when only the metamorphic portion of the sequence is considered.

pdb A	pdb B	Similarity	pdb A	pdb B	Similarity
1CEEB	2K42A	81.4	3IFAA	5ET5A	99.7
1DZLA	5KEQF	99.2	3J7VG	3J7WB	100
1EBOE	5FHCJ	52.0 (63.8)	3J97M	1XTGB	100
1FZPD	2FRHA	100	3J9CA	3Q8FA	100
1G2CF	5C6BF	83.7	3KUYA	5C3IF	98.5
1H38D	1QLNA	100	3L9QB	4RR2D	99.0
1HTMB	5HMGB	99.3	3LOWA	3M1BF	100
1IYTA	2NAOF	100	3MEEA	4B3OB	99.1
1JFKA	2NXQB	100	3MKOA	5INEA	92.0
1JTIB	1OVAA	99.5	3T5OA	4A5WB	100
1K0NA	1RK4B	99.2	3TP2A	5LJ3M	98.7
1KCTA	3T1PA	98.1	3UYIA	3V0TA	99.4
1MBYA	4YYPA	93.1	3VO9B	3VPAD	100
1MIQB	1QS8B	99.7	3ZWGN	4TSYD	100
1MNMC	1MNMD	100	3ZXGB	5EC5P	97.7
1NOCA	1QOMB	100	4AALA	4AANA	100
1NQDA	1NQJB	99.2	4AE0A	4OW6B	99.8
1NRJB	2GEDB	77.7	4CMQB	4ZT0C	99.9
1QB3A	3QY2A	99.1	4DXRA	4DXTA	99.0
1REPC	2Z9OB	99.2	4GQCB	4GQCC	100
1RKPA	2H44A	99.7	4JPHB	5HK5H	100
1SVFC	4WSGC	98.4	4M4RA	4W50B	98.3
1UXMK	2NAMA	99.3	4N9WA	4NC9C	100
1WP8C	5EJBC	53.9 (54.5)	4O01A	4O0PA	100
1WYYB	5WRGA	70.5	4PYIA	4PYJA	100
1X0GA	1X0GB	100	4Q79F	4UV2D	99.6
1XEZA	3O44A	100	4QHFA	4QHHA	100
1XJTA	1XJUB	100	4RMBA	4RMBB	100
1XNTA	3LQCA	99.5	4RWNA	4RWQB	100
1ZK9A	3JV6A	100	4TWAA	4YDQB	100
2A73B	3L5NB	100	4XWSD	4Y0MJ	99.6
2AXZA	2GRMB	99.7	4YHDG	7AHLE	99.3
2BZYB	2LQWA	100	5AOEB	5LY6B	98.3
2C1UC	2C1VB	100	5B3ZA	5BMYA	99.0

2CE7C	3KDSG	99.8	5C1VA	5C1VB	100
2HDMA	2N54B	95.7	5F3KA	5F5RB	100
2JMRA	4J3OF	100	5I2MA	5I2SA	100
2K0QA	2LELA	100	5JZHA	5JZTG	99.8
2KKWA	2N0AD	100	5L35D	5L35G	100
2LCLA	2OUGC	93.9	5SUZA		
2LEJA	2LV1A	100	3J9ED		
2LEPA	4HDDA	87.0	3TKAA		
2LHCA	2LHDA	98.2	4HLSA		
2MWFA	2NNTA	93.8	4OV8A		
2P3VA	2P3VD	100	4PMKA		
2PBKB	3NJQA	100	1S5PA		
2QKEA	5JYTA	90.6	2MZ7A		
2QQJA	4QDSA	100	2N4OA		
2UY7D	5FLUE	99.4	2KTMA		
2VFXL	3GMHL	93.2	2LE3A		
2WCDX	4PHQA	98.7	2LSHA		
3EJHA	3M7PA	93.5	2KXOA		
3EWSB	3G0HA	100	2X9CA		
3HDEA	3HDFA	99.3	3GAXA		

Supplementary Table S2. The monomorphic dataset. RMSD and SS penalty columns indicate maximum values that are taken over all of the child PDB IDs.

pdb ID	# of child pdb	Year	RMSD (Å)	SS Penalty
1K3YA	53	2001	1.5	9
2QWXA	67	2007	0.8	8
1UZVA	62	2004	2.1	2
2INCC	37	2006	1.3	3
2FKZA	60	2006	1.8	4
2J96A	43	2006	1.8	6
1MVQA	124	2002	1.8	3
1E85A	51	2000	1	3
1MQOA	46	2002	1.8	6
3BWHA	90	2008	2.2	7
1UGXA	49	2003	2.4	0
1KT6A	32	2002	2.1	9
3E86A	33	2008	1.3	4
1T56A	75	2004	2	7
1UBQA	567	1987	1.2	2
1YCKA	37	2004	1.3	7
1J4T	45	2001	1.7	8
3CHBD	58	1998	2.1	3
2DSCA	53	2006	1.2	3
3D80A	93	2008	2	9
1RYPH	306	1997	2.1	8
2FDJA	34	2005	1.9	5
1N5NA	34	2002	2.2	3
2BBKL	41	1993	0.9	4
3EF4A	40	2008	2.4	7
1GH0A	46	2000	1.8	5
1KLIL	34	2001	1	3
2CHHA	47	2006	2.1	0
1XEOA	34	2004	2.2	4
1PMYA	40	1994	2.1	7
2BURB	56	2005	1.1	9
2BZ6L	30	2005	1.5	3

1KMVA	91	2001	2	8
1XMEB	36	2004	1	9
8DFRA	93	1989	2	8
1OPSA	44	1997	1.6	8
1PHNA	46	1995	1.8	5
1PAZA	40	1988	2.4	6
2JETB	41	2007	1.2	3
2GDGA	95	2006	1.3	6
1JOTA	36	1997	2.4	0
1KM4A	114	2001	1.7	6
3EIKA	38	2008	2.3	4
3ELNA	64	2008	1.1	3
2OQAA	90	2007	2.2	7
2A06F	60	2005	2.2	9
2DYRI	59	2006	1.8	6
1K55A	110	2001	1.8	6
3D6MA	32	2008	1.3	7
2UU8A	122	2007	1.8	3
3ERXA	40	2008	2.1	8
3PCCB	52	1997	1.1	8
1KPFA	50	1997	1	5
4UBPA	48	1999	1.4	9
3DR9A	48	2008	1.2	8
2BMOB	31	2005	1.2	5
1CPCA	46	1990	1.8	5
3F0UX	55	2008	2.4	3
1UIZA	94	2003	1.3	6
3DJHA	94	2008	1.3	6
1EJXB	31	2000	0.8	4
2Z8AA	68	2007	1.4	8
2JE7A	124	2007	1.8	4
2IH3C	35	2006	1.8	5
3DHIC	37	2008	1.3	4
2BV4A	47	2005	2.1	0
1JJTA	35	2001	1.9	7
2BC3A	33	2005	1.1	3
1O7NB	31	2002	1.2	4

2RK3A	65	2007	0.9	5
1WXCB	32	2005	0.9	3
1TWFK	153	2004	2.45	7
253LA	577	1997	1.8	9
3SEBA	57	1997	2.4	9
7FD1A	32	1998	0.7	5
1ZBFA	87	2005	1.3	5
2UYZA	39	2007	1.6	2
1JBOA	43	2002	1.8	5
2VMLA	43	2008	1.8	6
1YPHC	48	2005	1.1	3
1MRJA	90	1994	2.2	7
2GJDA	39	2006	1.6	2
3E2CA	65	2008	2.2	7
1X8QA	54	2004	1.6	5
2NN8A	58	2006	0.9	0
1SLTA	42	1993	2.4	3
1F99A	46	2000	1.8	5
1IIUA	32	2001	2.1	8
1RVEA	31	1992	2.1	8
2BV8A	46	2005	1.9	5
1A53A	35	1998	2.4	8
1AB9B	54	1997	0.9	3
1AHCA	90	1994	2.2	9
1AUNA	144	1997	1.6	8
1BIOA	35	1998	1.6	6
1CEXA	55	1997	2	8
1ELTA	122	1995	2.3	9
1FIPA	33	1994	1.9	3
1FONA	122	1996	2.3	9
1FUSA	116	1993	2.1	8
1GFLA	276	1996	0.9	3
1GK8I	33	2001	1.5	7
1HJ8A	714	2001	2	6
1IAUA	36	2001	2.2	7
1J7DB	36	2001	2.1	4
1KEQA	810	2001	1.4	7

1L2HA	33	2002	2	6
1LM4A	37	2002	1.5	5
1LQYA	37	2002	1.4	5
1NN6A	36	2003	2.2	8
1QMJA	34	1999	2.4	2
1RYPL	262	1997	1.7	6
1T32A	36	2004	2.2	6
1VF1A	53	2004	1.8	9
1W6NA	34	2004	2.4	3
1WDDS	33	2004	1.5	6
1Z3QA	142	2005	1.6	7
2AWKA	302	2005	1.3	6
2DKOB	80	2006	2.1	5
2DYRF	45	2006	1.3	2
2DYRG	45	2006	1.4	4
2HBTA	33	2006	1.9	6
2HFTA	41	1995	1.9	6
2JKHA	69	2008	1.6	5
2O7MA	33	2006	1.3	9
2OGQA	52	2007	2.2	9
2OV0A	36	2007	1.7	3
2PGZA	74	2007	1.9	5
2UX7A	40	2007	2.1	4
2VHKA	142	2007	1.6	7
2VLQA	38	2008	1.8	8
2W69A	78	2008	2.3	8
3B44A	32	2007	2	9
3D6MB	32	2008	1.4	7
3DUIA	34	2008	2.4	2
3RP2A	36	1984	2.2	6
